# *Lactiplantibacillus* plantarum NKK20 Increases Intestinal Butyrate Production and Inhibits Type 2 Diabetic Kidney Injury through PI3K/Akt Pathway

**DOI:** 10.1155/2023/8810106

**Published:** 2023-12-23

**Authors:** Xiaohong Sun, Yue Xi, Man Yan, Chang Sun, Jianjun Tang, Xueyun Dong, Zhengnan Yang, Liang Wu

**Affiliations:** ^1^Department of Clinical Laboratory, Yizheng Hospital, Nanjing Drum Tower Hospital Group, Yizheng 210008, China; ^2^Medical Laboratory Department, Huai'an Second People's Hospital, Huai'an 223022, China; ^3^Department of Clinical Laboratory, Zhenjiang City Central Blood Station, Zhenjiang 212399, China; ^4^Department of Laboratory Medicine, School of Medicine, Jiangsu University, Zhenjiang 212013, China

## Abstract

Nephropathy injury is a prevalent complication observed in individuals with diabetes, serving as a prominent contributor to end-stage renal disease, and the advanced glycation products (AGEs) are important factors that induce kidney injury in patients with diabetes. Addressing this condition remains a challenging aspect in clinical practice. The aim of this study was to explore the effects of *Lactiplantibacillus plantarum* NKK20 strain (NKK20) which protects against diabetic kidney disease (DKD) based on animal and cell models. The results showed that the NKK20 can significantly reduce renal inflammatory response, serum oxidative stress response, and AGE concentration in diabetic mice. After treatment with NKK20, the kidney damage of diabetic mice was significantly improved, and more importantly, the concentration of butyrate, a specific anti-inflammatory metabolite of intestinal flora in the stool of diabetic mice, was significantly increased. In addition, nontargeted metabolomics analysis showed a significant difference between the metabolites in the mouse serum contents of the NKK20 administration group and those in the nephropathy injury group, in which a total of 24 different metabolites that were significantly affected by NKK20 were observed, and these metabolites were mainly involved in glycerophospholipid metabolism and arachidonic acid metabolism. Also, the administration of butyrate to human kidney- (HK-) 2 cells that were stimulated by AGEs resulted in a significant upregulation of ZO-1, Occludin, and E-cadherin gene expressions and downregulation of *α*-SMA gene expression. This means that butyrate can maintain the tight junction structure of HK-2 cells and inhibit fibrosis. Butyrate also significantly inhibited the activation of PI3K/Akt pathway. These results indicate that NKK20 can treat kidney injury in diabetic mice by reducing blood glucose and AGE concentration and increasing butyrate production in the intestine. By inhibiting PI3K pathway activation in HK-2 cells, butyrate maintains a tight junction structure of renal tubule epithelial cells and inhibits renal tissue fibrosis. These results suggest that NKK20 is helpful to prevent and treat the occurrence and aggravation of diabetic kidney injury.

## 1. Background

Diabetic kidney disease (DKD) is one of the main causes of end-stage nephropathy [1]. Diabetes is the most common metabolic disease in the world, and the incidence is still increasing year by year, and there is a significant trend of younger age [2]. DKD represents the primary complication associated with diabetes. Currently, there exists a substantial global population of individuals affected by DKD, thereby rendering the prevention and treatment of this condition a challenging undertaking. The etiology of DKD is intricate, and recent research has revealed that the elevated presence of advanced glycation end products (AGEs) in the bloodstream of individuals with diabetes plays a significant role in the development of this renal complication [3]. AGEs are irreversible glycosylation end products produced by enzymolysis-independent reactions between reduced sugars and a group of different substrates, including arginine and lysine residues in proteins, amino groups in phospholipids, and guanylic acid in DNA [4]. In addition, AGEs *in vivo* may also come from the autoxidation reaction during the process of glucose decomposition or gluconeogenesis [5]. Under normal circumstances, AGEs are formed in the body at a slow rate and can be cleared by the kidney [6]. However, in the context of hyperglycemia, the body's synthesis rate of AGEs is significantly enhanced, surpassing the kidney's capacity for clearance, thereby leading to the substantial accumulation of AGEs [7]. High concentrations of AGEs in the body can induce inflammation in various organs [8]. When AGEs accumulate in large quantities in the kidney, it will cause damage to the kidney structure and loss of function, leading to DKD and even uremia [9]. Therefore, AGEs are currently recognized as a potential indicator of kidney injury.

The clinical studies have also shown that the short-chain fatty acids (SCFAs) are highly correlated with the improvement of symptoms and complications in type 2 diabetes (T2DM) [10–12]. SCFAs are generated through the process of fermentation of indigestible dietary fiber by specific anaerobic bacteria located in the colon. These SCFAs primarily consist of acetic acid, propionic acid, and butyric acid, along with other organic acids possessing fewer than six carbon atoms [13]. Butyrate, an SCFA, is primarily produced in the gastrointestinal tract through the fermentation of dietary fiber. Although its main origin is in the gut, it can also enter the bloodstream and contribute to the development of diseases associated with inflammation and immunity, including inflammatory bowel disease, asthma, and arthritis [14–16]. Previous experiments revealed that sodium butyrate (NaB) could alleviate the oxidative stress and inflammation caused by AGEs by regulating cellular metabolism [17]. Therefore, butyrate may play an important role in improving DKD.

Existing clinical studies can confirm that a variety of probiotics can effectively inhibit the inflammatory response in diabetic patients and can significantly reduce the risk of kidney damage in elderly diabetic patients [18, 19]. Some clinical studies have indicated that soy milk containing *Lactiplantibacillus plantarum* can improve kidney function in T2DM patients with nephropathy [20, 21]. In a recent study, butyrate-producing *Lactiplantibacillus plantarum* can ameliorate hyperglycaemia in streptozotocin-induced diabetic mice [22]. *Lactiplantibacillus plantarum* NKK20 strain (NKK20) is a newly isolated strain from the intestines of healthy humans that has been deposited in the China Typical Culture Preservation Center under the preservation number CCTCC NO: M2020596. Previous studies have shown that this NKK20 strain has the ability to promote the production of SCFAs, including butyrate, in the colon contents, and confers strong hypolipidemic and anti-inflammatory effects [23]. These results suggest that butyrate-producing NKK20 may be an effective dietary means to improve DKD. Nevertheless, the protective effect of NKK20 on the renal injury in DKD remains unclear. The pathway, which NKK20 ameliorates renal injury in DKD, has not been clarified.

We evaluated the effect of NKK20 on improving blood glucose and AGE levels and kidney injury based on a mouse model of diabetic kidney injury. Basing on the human kidney- (HK-) 2-cell model, the treatment mechanism of AGE-induced kidney injury by butyrate was studied. These studies can provide theoretical support for probiotic therapy in patients with DKD.

## 2. Materials and Methods

### 2.1. Animal and DKD Model

Thirty 6-week-old male ICR mice were purchased from Wukong Biological Company (Nanjing, China) and raised in the Animal Laboratory Center of Jiangsu University with barrier system. Conventional mouse diet and high-fat diet (HFD) were purchased from Jiangsu Xietong Research and Bio-Engineering Co., Ltd. (Nanjing, China). ICR mice were randomly divided into 3 groups, including the control group (NC), diabetic kidney disease model group (DKD), and HFD plus NKK20 group (NKK20), with 10 mice in each group. The mice in the NC group were fed with conventional feed until the end of the experiment. Mice in the DKD group and NKK20 group were fed HFD, and diabetic nephropathy was induced by intra-abdominal injection of streptozotocin (50 mg/kg) for 5 consecutive days starting from week 5. At the beginning of the experiment, mice in the NKK20 group were given NKK20 by oral administration, and each mouse was given 1 × 10^7^ viable bacteria per day. The blood glucose concentration in the tail vein in the fasting state of mice was detected for 3 consecutive days, and the diabetic nephropathy model was considered successful when the fasting blood glucose (FBG) level was ≥11.1 mmol/L. The HFD was continued for 6 weeks, and body weight and FBG were monitored during the experiment. The mice were killed at the end of the 9th week, and serum and kidney cortex were collected for follow-up study.

### 2.2. HK-2 Cell Culture and Treatment

HK-2 cells (ATCC® CRL-2190™, Rockville, MD, USA, a kindly gift from professor Hui Qian, School of Medicine, Jiangsu University) were cultured in RPMI 1640 medium containing 10% fetal bovine serum at 37°C and 5% CO_2_. HK-2 cells were inoculated in a 6-well cell culture plate with a density of 1 × 10^6^ cells for 24 hours for the experiment. Cell experiments are divided into five groups: normal control group (NC), AGE stimulation group (AGEs), AGEs+low dose NaB (100 *μ*mol/L) group (L-NaB), AGEs+medium dose (200 *μ*mol/L) group (M-NaB), and AGEs+high dose NaB (400 *μ*mol/L) group (H-NaB). The cells in the NC group were cultured with conventional cell culture medium without adding NaB and AGEs. In the NaB intervention groups, the different concentrations of NaB were incubated in advance for 24 h, and then the cells in the AGEs group and NaB groups were treated with 400 *μ*g/mL of AGEs to induce inflammatory response for 24 h. Cell cultures were collected at the end of the experiment for total RNA extraction and western blotting assays.

### 2.3. qRT-PCR Assay

Total RNA of mouse kidney tissues and HK-2 cells were extracted by RNA-easy isolation reagent (Vazyme Biotech Co., Ltd., Nanjing, China) in qRT-PCR experiment. By HiScript III 1st strand cDNA synthesis kit (+gDNA wiper) (Vazyme Biotech Co., Ltd., Nanjing, China), the expression of related genes in kidneys and HK-2 cells of mice was detected by AceQ Universal SYBR qPCR Master Mix (Vazyme Biotech Co., Ltd., Nanjing, China). PCR primers were synthesized by GENEWIZ (Suzhou, China). qPCR reaction conditions are as follows: 95°C for 30 s, 95°C for 5 s, and 60°C for 30 s, a total of 40 cycles. The results of qPCR were expressed by Ct value, the GAPDH gene was used as the internal reference, and the relative expression of gene mRNA was calculated by 2^−ΔΔCt^ calculation method. The primer sequences are shown in Table 1.

### 2.4. Western Blotting Assay

At the end of the experiment, HK-2 cells were collected, the cell culture medium was discarded, and the residual cell culture medium was removed by washing with phosphate buffer precooled on ice for 3 times. RAPI lysate (Beyotime, Nanjing, China) was added to the cells and lysed on ice for 30 min. The total protein in the lytic supernatant was collected, and the protein concentration was detected by BCA protein quantification kit (Beyotime, Nanjing, China). All samples were separated by SDS-PAGE electrophoresis. After electrophoresis, constant electrophoresis model was transferred to polyvinylidene fluoride (PVDF). The transferred PVDF membrane was soaked and sealed in 5% skim milk powder (Boster Biological Technology Co., Ltd., Wuhan, China) for 2 h and then combined with various moderately diluted antibodies for overnight incubation (ABclonal Technology Co., Ltd., Wuhan, China). These include *α*-SMA (1 : 2000), E-cadherin (1 : 2000), AkT (1 : 1000), p-AkT (1 : 500), PI3K (1 : 1000), p-PI3K (1 : 500), and actin (1 : 10000). After incubation, the unbound antibodies were removed by washing with TBST buffer (Vazyme Biotech Co., Ltd., Nanjing, China) for 3 times and then incubated with HRP-labeled goat anti-rabbit IgG (Boster Biological Technology Co., Ltd., Wuhan, China) at room temperature for 1 h. After the incubation of the antibody was completed, the ECL color-developing solution (Vazyme Biotech Co., Ltd., Nanjing, China) was added to expose.

### 2.5. Hematoxylin and Eosin (H&E) and Masson Staining

Mouse renal parenchymal tissue was immersed in 4% paraformaldehyde and fixed for 48 hours before staining. Mouse renal parenchymal tissue was sliced by paraffin embedding, dewaxed, dehydrated in ethanol with gradient concentration, and finally used for staining. H&E staining was used to observe cell morphology in renal parenchyma. The degree of renal parenchymal fibrosis was observed by Masson staining. In diabetic nephropathy, fibroblasts in the kidney were activated and transformed into myofibrocytes, which further synthesized and secreted a large number of collagen fibers that were difficult to degrade, and finally caused the accumulation of extracellular matrix resulting in renal interstitial fibrosis. After Masson staining, the myofibrillar fibers were red, and the collagen fibers were green. The Image-Pro Plus 6.0 software was used to scan the Masson staining results and score according to the size of the green area.

### 2.6. ELISA Assay and Enzyme Activity Detection

AGE concentration in the serum of mice was detected by ELISA kit (ZCI Bio, Shanghai, China). The superoxide dismutase (SOD) activity assay kit and malondialdehyde (MDA) content assay kit were purchased from the Jiancheng Institute of Bioengineering (Nanjing, China). The testing procedure is carried out according to the kit operating instructions.

### 2.7. Nontargeted Metabolomics Analysis

The mouse serum samples were thawed at 4°C, 60 *μ*L of serum was obtained, and acetonitrile was added with the volume ratio of 1 : 4 to remove protein. Then the mixture was violently shaken for 1 min and centrifuge at 4°C at 13500 rpm for 15 min. The supernatant was filtered through a filtration centrifuge tube with a pore size of 0.22 *μ*m to remove particulate matter, and 200 *μ*L of the filtered sample was used for nontargeted metabolomics analysis by UPLC-Q-TOF-MS in Wekemo Tech Group Co., Ltd. (Shenzhen, China). The mobile phases were water containing 0.1% formic acid (phase A) and acetonitrile containing 0.1% formic acid (phase B) at the flow rate of 0.3 mL min-1. The gradient elution conditions were 0-7 min, 55% B; 7-9 min, 55%-80% B; and 9-15 min, 80%-100% B. Mass spectrum conditions are as follows: ESI ionization source was used, the ion source temperature was 120°C, the drying temperature was 225°C, the flow rate was 5 L min^−1^, the atomizer pressure was 20 psi, the sheath temperature was 400°C, the sheath gas flow rate was 12 L min^−1^, and the nozzle voltage was 500 V. Scanning range was m/z 20^−1^ 700 in positive ion mode with a capillary voltage of 3 500 V and in negative ion mode with a capillary voltage of 4 000 V. The chromatogram of serum samples in each group was performed by Markerview 2.1 (AB SCIEX, Massachusetts, USA) software. The chromatographic peak was extracted, and the peak area was normalized. The normalized data were analyzed by principal component analysis (PCA) to observe the changes of metabolic profile in each group of mice. SIMCA-P v11.5 (Umetrics, Umea, Sweden) software was used to analyze the content results of the detected compounds by orthogonal partial least squares discriminative analysis (OPLS-DA), and a VIP-plot was obtained that could reflect the contribution rate between groups. Based on OPLS-DA model variables, differential metabolites were screened according to VIP > 1 and *P* < 0.05 rule. MetaboAnalyst 3.0 (http://www.metaboanalyst.ca/) was used for pathway analysis of the obtained differential metabolites, and the biological significance of the differential metabolites was further analyzed by combining HDMB and KEGG databases. Finally, the potential biomarkers and related metabolic pathways associated with NKK20 in the treatment of diabetic kidney injury were identified.

### 2.8. GC-MS Detection of Butyric Acid

The concentration of butyric acid in mouse feces was determined by Wekemo Tech Group Co., Ltd. (Shenzhen, China). The concentration of butyric acid was determined by gas chromatography. The specific method is as follows: 0.1 g mouse stool sample was thoroughly mixed with 1 200 *μ*L sterilized distilled water, and then 50 *μ*L (50%) concentrated sulfuric acid was added to the mixture for acidification. The concentration of butyric acid was determined by gas chromatograph using diethylbutyric acid as internal standard.

### 2.9. Statistical Analysis

Statistical software SPSS 20.0 was used for data analysis. Measurement data are expressed as mean ± standard deviation. One-way ANOVA was used for multigroup comparison, and LSD method was used for pair-to-group comparison. *P* < 0.05 was considered statistically significant.

## 3. Results

### 3.1. Effects of NKK20 on Body Weight and Blood Glucose in DKD Mice

**Figure 1:**
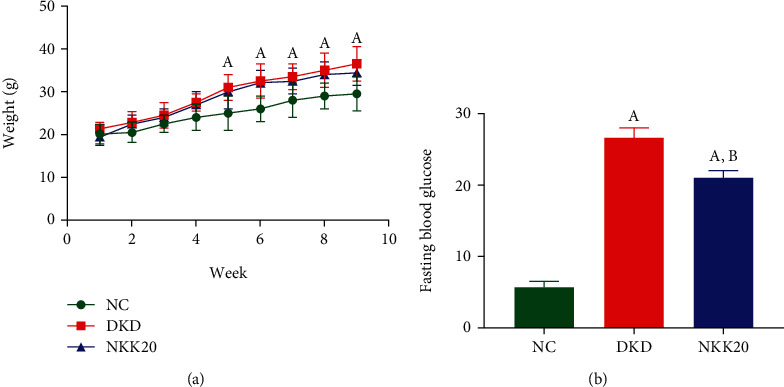
Body weight and blood glucose level in mice. (a) Body weight change. (b) Fasting blood glucose levels. (A) Compared with the NC group, *P* > 0.05. (B) Compared with the DKD group, *P* > 0.05.

As can be seen from Figure 1(a), the body weight of mice in the DKD group and NKK20 group w*as* significantly higher than that in the NC group from week 5 (*P* < 0.05), while there was no significant difference in body weight between the DKD group and NKK20 group (*P* > 0.05). As shown in Figure 1(b), when the experiment was discontinued, the fasting blood glucose (FBG) of mice in the DKD group and NKK20 group w*as* significantly higher than that in the NC group, and the FBG level in the DKD group was significantly higher than that in the NKK20 group (*P* < 0.05).

### 3.2. Effects of NKK20 on Inflammatory Responses in Diabetic Mice

**Figure 2:**
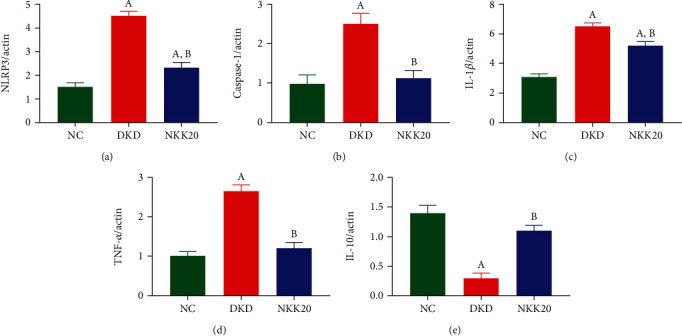
The expression of inflammatory factors in the spleen of mice detected by qPCR assay. (a) NLRP3, (b) caspase-1, (c) IL-1*β*, (d) TNF-*α*, and (e) IL-10. (A) Compared with the NC group, *P* < 0.05. (B) Compared with the DKD group, *P* < 0.05.

The results of qPCR assay showed that the expressions of NACHT, LRR, and PYD domain-containing protein 3 (NLRP3), caspase-1, and interleukin-1beta (IL-1*β*) in the kidney tissues of DKD model mice were significantly higher than those of the NC group, while these expressions after oral administration of NKK20 were significantly decreased (*P* < 0.05) (Figures 2(a)–2(c)). Compared with the NC group, the proinflammatory cytokine tumor necrosis factor-alpha (TNF-*α*) in the kidney tissues in the DKD group was significantly increased (*P* < 0.05), while the anti-inflammatory cytokine interleukin-10 (IL-10) was significantly decreased (*P* < 0.05) (Figures 2(d) and 2(e)). Compared with the DKD group, the TNF-*α* expression was significantly decreased (*P* < 0.05), while the IL-10 expression was significantly increased (*P* < 0.05) in the NKK20 group.

### 3.3. Effects of NKK20 on SOD, MDA, AGEs, and Butyrate Levels in Diabetic Mice

**Figure 3:**
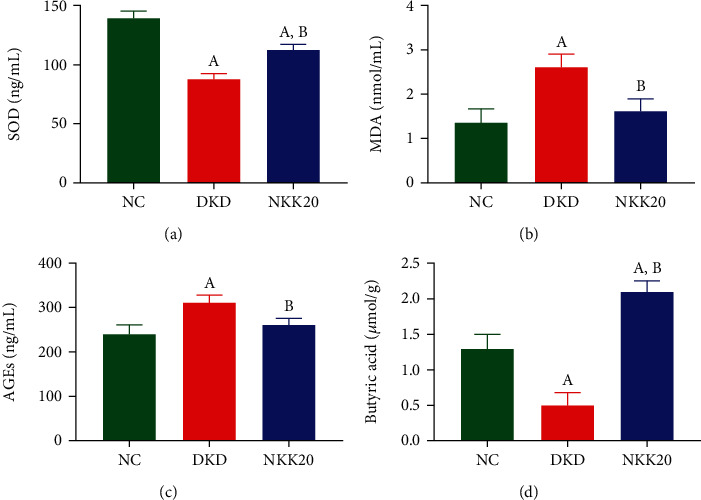
The levels of SOD, MDA, and AGEs in serum and the concentration of butyrate in stool in diabetic mice. (a) SOD content, (b) MDA content, (c) AGEs level, and (d) butyrate content. (A) Compared with the NC group, *P* < 0.05. (B) Compared with the DKD group, *P* < 0.05.

The SOD content in serum of DKD mice was significantly lower than those in the NC group, and the MDA content in serum of DKD mice was significantly higher than those in the NC group (*P* < 0.05). Compared with the DKD group, SOD content in the NKK20 group was significantly increased, and MDA concentration was significantly decreased (*P* < 0.05) (Figures 3(a) and 3(b)). The serum concentration of AGEs in DKD mice was found to be significantly higher compared to the NC group. Conversely, the serum concentration of AGEs in mice that received oral administration of NKK20 exhibited a significant decrease when compared to the DKD group (Figure 3(c)). GC-MS assay results showed that the fecal butyrate concentration of DKD mice was significantly lower than that of the NC group, while the fecal butyrate concentration of DKD mice was significantly higher after oral administration of NKK20 (*P* < 0.05) (Figure 3(d)).

### 3.4. Effects of NKK20 on Renal Injury in Diabetic Mice

**Figure 4:**
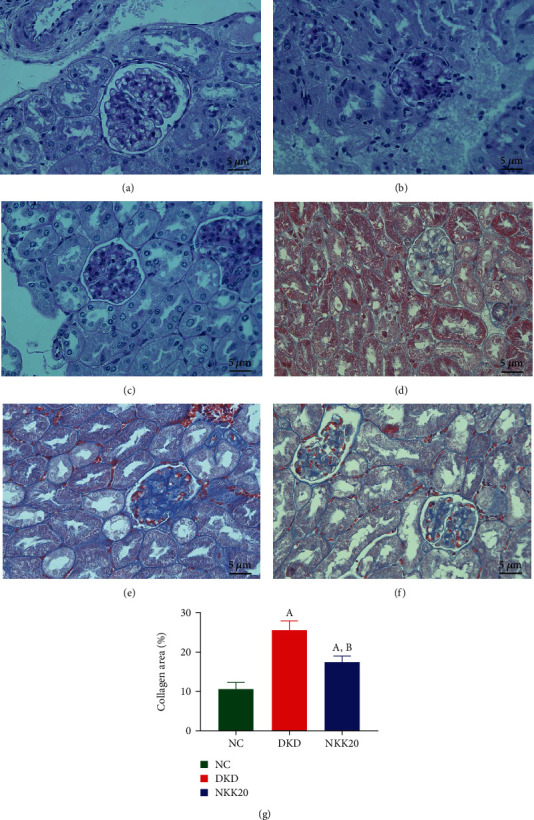
H&E staining (a–c) and Masson staining (d–f) in mouse kidney tissues. (a) NC group, (b) DN group, (c) NKK20 group, (d) NC group, (e) DKD group, and (f) NKK20 group. (g) Compared with the NC group, *P* < 0.05 (A); compared with the DKD group, *P* < 0.05 (B).

Hematoxylin and eosin (H&E) staining showed that the glomeruli and renal tubules of the NC group mice were regular in shape; and the epithelial cells of renal tubules were arranged neatly, with complete morphology and uniform cytoplasm. In the DKD group, the renal tubules exhibited focal degeneration and atrophy, accompanied by a slight thickening of the glomerular basement membrane and mesangial hyperplasia (Figures 4(a)–4(c)). Importantly, the above pathological conditions were significantly improved in the NKK20 intervention group. The collagen fibers within the kidney tissue exhibited a blue hue following Masson staining. Notably, the DKD group displayed a substantial increase in the blue area within the mesangial region and basement membrane, when compared to the NC group. Conversely, the NKK20 group demonstrated a reduction in the area of blue collagen within the glomerulus and renal tubule interstitium, in comparison to the DKD group (*P* < 0.05) (Figures 4(d)–4(f)).

### 3.5. Effects of NKK20 on Serum Endogenous Metabolites in Diabetic Mice

**Figure 5:**
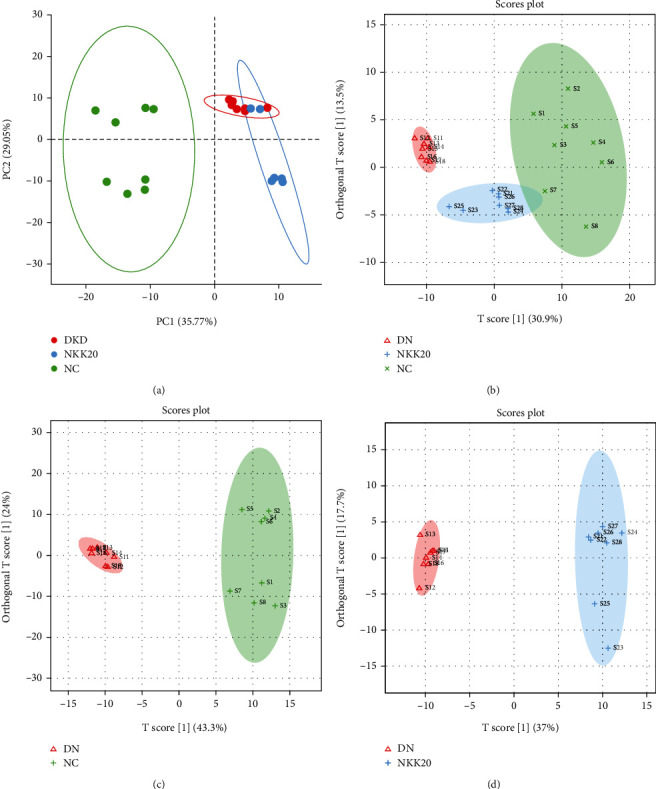
Serum nontargeted metabolomics was analyzed by PCA and OPLS-DA. (a) PCA and (b–d) OPLS-DA.

The serum metabolites of each group were analyzed by UPLC-Q-TOF-MS, and the serum samples were scanned in negative mode (ESI^−^), which showed some differences in the metabolite content of each group. To obtain metabolic differences between each group, multivariate statistical analysis was performed. The unsupervised PCA score map showed that the NC and DKD groups significantly clustered into two categories (Figure 5(a)), indicating significant differences in serum endogenous metabolites between the two groups. In order to further evaluate the effectiveness of NKK20 in the treatment of DKD, supervised OPLS-DA analysis was performed in each group, and it was found that the serum metabolic profile of mice was significantly separated between the NC and DKD groups and between the DKD and NKK20 groups. Moreover, the sample points of the serum of mice in the NC and NKK20 groups were closer to each other, indicating that NKK20 affected the serum metabolic profile of DKD mice, making it tend to normal mice (Figures 5(b)–5(d)). OPLS-DA is a supervised forecasting model. In ESI^−^ mode, *R*^2^*X* = 0.444, *R*^2^*Y* = 0.915, and *Q*^2^ = 0.817, indicating that the model has good forecasting ability.

**Figure 6:**
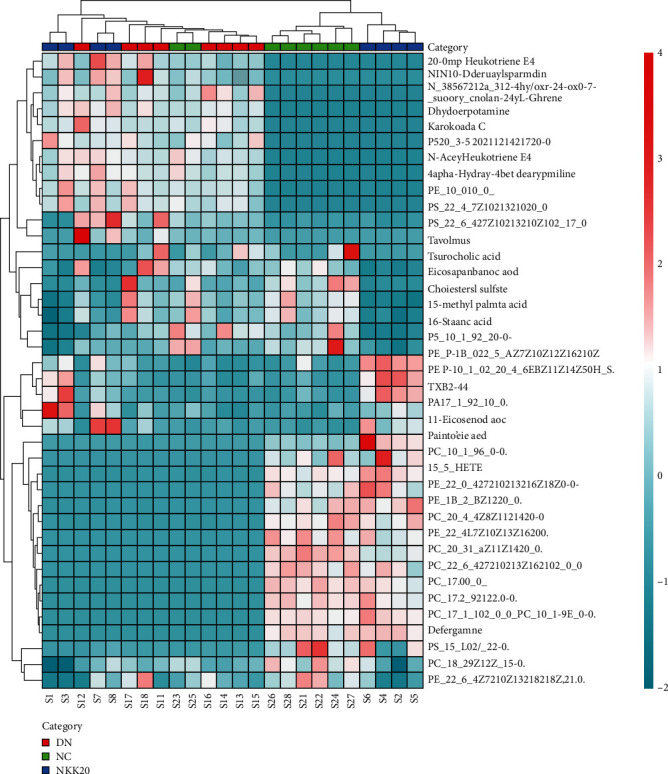
Heat map of mouse serum metabolites cluster.

**Figure 7:**
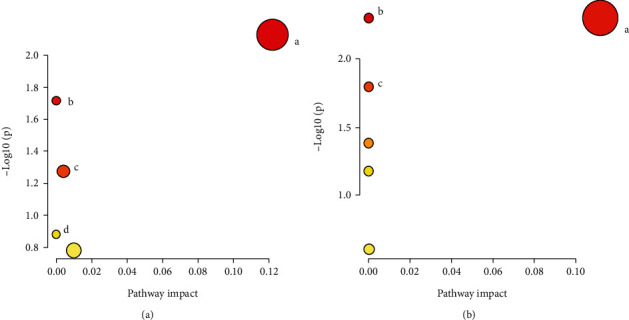
Enrichment map of metabolic pathways. (a) NC group vs. DKD group; (b) DKD group vs. NKK20 group. (a) Glycerol phospholipid metabolic pathway (A); linoleic acid metabolic pathway (B); glycosylphosphatidylinositol- (GPI-) anchored biosynthetic pathway (C); arachidonic acid metabolic pathway (D). (b) Glycerol phospholipid metabolic pathway (A); arachidonic acid metabolic pathway (B); linoleic acid metabolic pathway (C).

Potential differential metabolites were screened using rules with VIP > 1 and *P* < 0.05, accurate m/z by first-order mass spectrometry, matching using the Mass Profiler Professional software ID Browser Identification function and METLIN metabolite database, looking for the possible structural formulas, and molecular formula matching with online databases such as Human Metabolome Database (http://www.hmdb.ca/) and KEGG (https://www.kegg.jp/kegg/). Then, comparing the secondary fragmentation information with the fragmentation information and literature in MassBank (http://www.massbank.jp/) and ChemSpider (https://www.chemspider.com/) databases, the metabolites were then identified. Finally, 16 serum potential biomarkers were selected between NC and DKD groups (Table 2), and 24 serum potential biomarkers were selected between the DKD and NKK 20 groups (Table 3 and Figure 6). Analysis of metabolic pathways using MetPA (https://www.metaboanalyst.ca/) MetPA is mainly based on KEGG metabolic pathway and HMDB database, combined with the results of pathway enrichment analysis and topology analysis, to select the metabolic pathways most relevant to the experiment. A total of three related metabolic pathways were selected using MetPA, including glycerophospholipid metabolism pathway, arachidonic acid metabolism pathway, and linoleic acid metabolism pathway (Figure 7).

### 3.6. NaB Upregulates Tight Junction Protein Expression and Suppresses Fibrosis in HK-2 Cells

**Figure 8:**
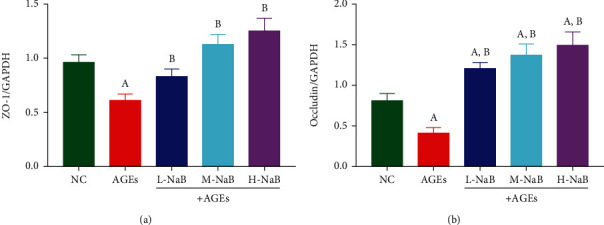
The expressions of ZO-1 and Occludin in HK-2 cells were determined by qPCR. (a) ZO1 mRNA level. (b) Occludin mRNA level. (A) Compared to the NC group, *P* < 0.05; (B) compared to the AGE group, *P* < 0.05.

**Figure 9:**
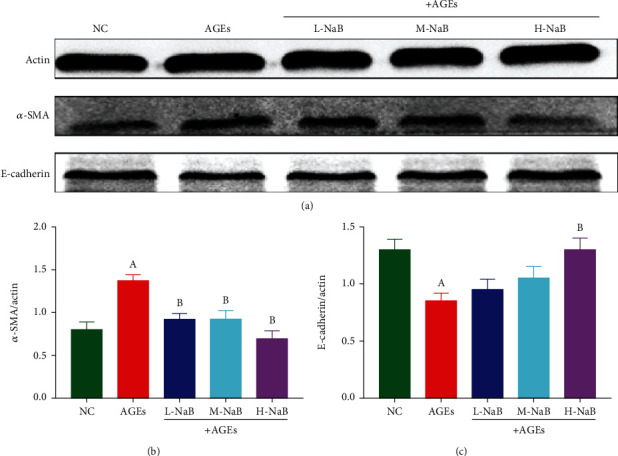
The expressions of *α*-SMA and E-cadherin in HK-2 cells were determined by western blotting assay. (a) Protein changes. (b) *α*-SMA. (c) E-cadherin. (A) Compared to the NC group, *P* < 0.05; (B) compared to the AGEs group, *P* < 0.05.

The mRNA expressions of the tight junction proteins ZO-1 and Occludin in HK-2 cells were determined by qPCR. Compared with the NC group, the mRNA expressions of ZO-1 and Occludin were significantly decreased in the AGEs group (*P* < 0.05); and after the treatment with the three concentrations of NaB, the mRNA expressions of ZO-1 and Occludin were significantly higher compared with the AGEs group (*P* < 0.05) (Figure 8). The expressions of *α*-SMA and E-cadherin were determined by western blotting in HK-2 cells. Compared with the NC group, the AGE stimulation had significantly more *α*-SMA expression and lower E-cadherin expression (*P* < 0.05). Compared with the AGE group, all three concentrations of NaB treatment significantly inhibited *α*-SMA expression (*P* all < 0.05), and only the cells in the H-NaB group significantly increased E-cadherin expression (*P* < 0.05) (Figure 9).

### 3.7. NaB Inhibits the Activation of PI3K-Akt Signaling Pathway in HK-2 Cells

**Figure 10:**
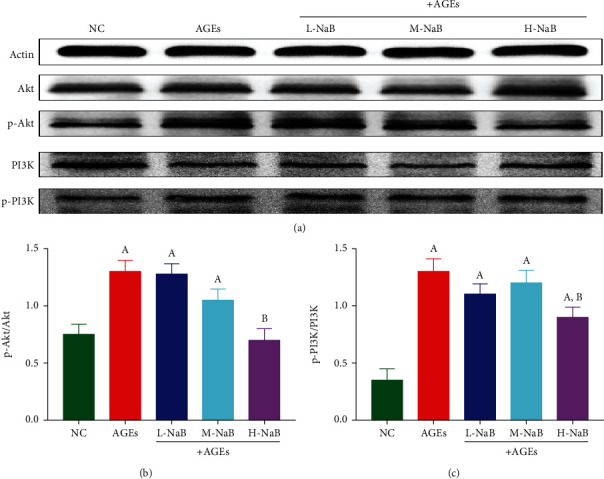
The expression of PI3K-Akt signaling pathway in HK-2 cells was determined by the western blotting assay. (a) Protein changes. (b) p-Akt/Akt. (c) p-PI3K/PI3K. (A) Compared to the NC group, *P* < 0.05; (B) compared to the AGE group, *P* < 0.05.

Phosphorylation of the phosphoinositide 3-kinase- (PI3K-) Akt signaling pathway was detected by the western blotting assay. Compared with the NC group, the protein expressions of p-Akt were significantly increased in the AGE group (*P* < 0.05); and after the treatment with the H-NaB, the protein expressions of p-Akt were significantly lower compared with the AGE group (*P* < 0.05) (Figures 10(a) and 10(b)). Compared with the NC group, the protein expressions of p-PI3K were significantly increased in the AGE group (*P* < 0.05); and after the treatment with the H-NaB, the protein expressions of p-PI3K were significantly lower compared with the AGE group (*P* < 0.05) (Figures 10(a) and 10(c)).

## 4. Discussion

DKD is one of the diabetic microvascular complications. The persistent hyperglycemia in the body of diabetic patients can lead to the metabolic abnormalities of some tissues and organs and then produce dysfunction and morphological changes, causing chronic complications of diabetes, which is one of the most common complications of diabetes [24, 25]. At present, the relatively accepted pathogenesis hypothesis of diabetic nephropathy is nonenzymatic glycochemistry, that is, under long-term hyperglycemia, glucose molecules undergo nonenzymatic glycosylation reactions with proteins *in vivo* to form irreversible AGEs, which then leads to the occurrence of kidney injury [26–28]. At the same time, studies have also confirmed that the progression of complications such as diabetic nephropathy can be significantly inhibited by inhibiting the glycosylation process [29–31].

The imbalance of intestinal flora is the direct cause of obesity, insulin resistance, diabetes, intestinal diseases, and cardiovascular metabolic diseases [32–34]. Recently, it has been recognized that changes in gut microbiota may also play an important role in the development of DKD, but the specific mechanism has not been defined, and therapies targeting intestinal microbiota are considered as a new approach for strategies against DKD [35]. The clinical studies have shown that insufficient dietary fiber intake and high intake of processed carbohydrates are major risk factors for diabetes, and dietary fiber intake is negatively correlated with the risk of diabetes [36, 37]. Intervention studies in humans have shown that increasing dietary fiber and whole grain intake can enhance gut microbial diversity, which not only helps the host regulate immune response and homeostasis but also participates in energy metabolism [38]. Dietary fiber that cannot be digested by the human body can be fermented by certain anaerobic microorganisms in the colon, and SCFAs are the main fermentation products [39]. SCFAs are a class of fatty acids with fewer than 6 carbon atoms, including formic acid (C1), acetic acid (C2), propionic acid (C3), butyric acid (C4), and valeric acid (C5), but the total amount of acetic acid, propionic acid, and butyric acid in the intestine accounts for more than 95% of all SCFAs, among which butyric acid has the strongest anti-inflammatory effect. Acetic acid can be formed from pyruvate via the acetyl-CoA pathway, propionic acid is mainly formed from succinic acid via the succinic acid pathway or from lactic acid via the acrylate pathway, and butyric acid is mainly formed from acetyl-CoA and butyryl-CoA as well as acetate and lactate [40, 41].

Butyrate can activate the genes that regulate the early development of the pancreas in embryonic stem cells to increase the differentiation of islet *β* cells and the expression of insulin coding genes, inhibit the apoptosis of islet *β* cells, and improve glucose homeostasis in diabetic rats through p38/ERK MAPK signaling pathway [42, 43]. Some scholars believe that butyrate may be the most promising chemical for the treatment of T2DM [44]. Our study showed that NKK20 could significantly increase the concentration of butyric acid in the stool of mice, significantly inhibit the activation of NLRP3 inflammasome and the secretion of TNF-*α* and other proinflammatory cytokines in the spleen of mice, and inhibit the oxidative stress response in diabetic mice and the inflammatory damage and fibrosis of kidney tissue. Because of its unpleasant odor, butyrate cannot be added to the human diet, and it is completely feasible to increase the production of butyrate in the gut by oral administration of *Lactiplantibacillus plantarum*.

Further metabolomic studies showed that NKK20 can inhibit diabetic kidney injury by regulating glycerin phospholipid metabolism and arachidonic acid metabolism. The essence of diabetic nephropathy is chronic inflammation [45, 46]. Glycerophospholipid metabolism and arachidonic acid metabolism are closely related to inflammation [47–50]. Inflammation is one of the most common pathological processes in all kinds of human diseases, and inflammation can lead to the disorder of lipid metabolism [51, 52]. Lipids are energy providers of organisms, participate in a large number of life activities, and have very important physiological functions, including maintaining cell membrane structure, energy storage, signal transduction, and carrier [53, 54]. Glycerin phospholipids are the main substances in cell membrane phospholipids, accounting for about 60% of lipid molecules. They have a variety of biological functions, and the disorder of their metabolic network can cause a variety of diseases, such as coronary heart disease, atherosclerosis, diabetes, obesity, cancer, brain injury, pain and inflammation, and Alzheimer's disease [55]. A systematic study of the dynamic changes in the molecular composition of glycerophospholipids is helpful to explain the molecular mechanism of disease pathogenesis [56, 57]. Arachidonic acid is a fatty acid lipid component, derived from the metabolism of glycerol phospholipids in lipid substances. Under pathological conditions such as inflammation and tumor, membrane phospholipids accelerate the release of arachidonic acid under the action of phospholipase A2 [58]. An excess of glycerophospholipids in the body can induce ER stress, which is associated with insulin resistance and T2DM [59]. When excessive glycerophospholipids are glycosylated in the body, oxidation sensitivity under hyperglycemia conditions will be increased, reactive oxygen species production will be increased, and inflammation and diabetic nephropathy will be further induced in the body [58]. In this study, we found that NKK20 can significantly reduce the contents of various glycerolipids in serum, which may be related to the improvement of diabetic kidney injury.

Based on the fact that oral administration of NKK20 strain significantly increased the production of butyrate in the colon of mice with diabetic nephropathy, we further cultured HK-2 cells *in vitro* to investigate the mechanism of butyrate inhibiting renal injury and fibrosis. Our results show that butyrate can increase the expression of the tight junction proteins ZO-1 and Occludin, improve the fibrosis level of HK-2 cells, and inhibit the phosphorylation of the PI3K/Akt signaling pathway. PI3K, also known as a lipid kinase, produces PIP3 to regulate the translocation of Akt as a second messenger to the plasma membrane [60]. The PI3K/Akt signaling pathway plays a key regulatory role in the occurrence and development of diabetic nephropathy, and the PI3K/Akt pathway is activated in renal tubular cells under diabetic conditions [61]. We speculate that inhibiting the activation of PI3K/Akt signaling pathway may be one of the mechanisms by which butyrate improves diabetic kidney injury, which needs further investigation.

A limitation of the present study is that the butyric acid responsible for the improvement on DN of NKK20 in mouse model was not identified. Therefore, the decisive role of butyric acid on DN warrants further research. Toll-like receptors (TLRs) as pattern recognition receptors play a key role in the proinflammatory process, and IL-1*β* and TNF-*α* are the primary downstream targets of TLR signaling [62, 63]. TLR inactivation by butyric acid-producing NKK20 may be attributed to the improvement on DN; however, this hypothesis requires further investigation.

## 5. Conclusion

In summary, NKK20 reduced kidney injury and fibrosis, oxidative stress, and inflammatory reactions in a diabetic kidney disease murine model. Moreover, glycerophospholipid metabolism and arachidonic acid metabolism were altered, and the concentrations of butyrate were increased. Furthermore, in an AGE-stimulated HK-2 cell model, butyrate can maintain tight junction and inhibit renal cell fibrosis and PI3K-Akt activation induced by AGEs. Our study shows that the increase of butyrate caused by NKK20 may be a key mechanism for reducing the development of diabetic kidney disease, and NKK20 may be used as a potential probiotic for the treatment and prevention of diabetic kidney disease.

## Figures and Tables

**Table 1:** qRT-PCR primer sequences.

Genes	Primer sequences (5′⟶3′)
*HK-2_GAPDH*	F: CATCACTGCCACCCAGAAGACTG R: ATGCCAGTGAGCTTCCCGTTCAG
*HK-2_ZO-1*	F: GAGCCTAATCTGACCTATGAACC R: TGAGGACTCGTATCTGTATGTGG
*HK-2_Occludin*	F: CTTCCAATGGCAAAGTGAATGAATGAC R: TACCACCGCTGCTGTAACGAG
*Mouse_GAPDH*	F: CATCACTGCCACCCAGAAGACTG R: ATGCCAGTGAGCTTCCCGTTCAG
*Mouse_IL-1β*	F: CCTGTCCTGCGTGTTGAAAGA R: GGGAACTGGGCAGACTCAAA
*Mouse_TNF-α*	F: AATGGCGTGGAGCTGAGA R: TGGCAGAGAGGAGGTTGAC
*Mouse_NLRP3*	F: AACAGCCACCTCACTTCCAG R: CCAACCACAATCTCCGAATG
*Mouse_Caspase-1*	F: GCACAAGACCTCTGACAGCA R: TTGGGCAGTTCTTGGTATTC
*Mouse_IL-10*	F: TCTCCGAGATGCCTTCAGCAGA R: TCAGACAAGGCTTGGCAACCCA

**Table 2:** Potential differential metabolites between the NC group and DKD group.

No	Compounds	Formula	Library ID	m/z	R/T	VIP	*P*	Change
1	15(S)-HETE	C20H32O3	HMDB03876	319.23	10.06	1.10	0.001	↓
2	PE(18 : 1(9Z)/22 : 6(4Z,7Z,10Z,13Z,16Z,19Z))	C45H76NO8P	LMGP01011315	770.52	10.72	1.16	0.001	↑
3	10,11-Dihydro-20-trihydroxy-leukotriene B4	C20H34O7	HMDB12503	385.22	8.43	1.28	0.001	↓
4	Taurochenodesoxycholic acid	C26H45NO6S	HMDB00951	498.29	6.98	1.08	0.001	↑
5	Stearic acid	C18H36O2	HMDB00827	283.26	13.07	1.21	0.001	↑
6	9,10-Epoxyoctadecenoic acid	C18H32O3	HMDB04701	591.46	12.58	1.21	0.001	↑
7	11-Eicosenoic acid	C20H38O2	HMDB34296	331.26	11.92	1.29	0.001	↓
8	PC(18 : 0/0 : 0)	C26H54NO7P	LMGP01050026	522.36	11.49	1.28	0.001	↓
9	3-beta-Hydroxy-4-beta-methyl-5-alpha-cholest-7-ene-4-alpha-carboxylate	C29H48O3	HMDB11662	489.36	11.73	1.33	0.001	↑
10	Prostaglandin F1a	C20H36O5	HMDB02685	337.24	11.56	1.29	0.001	↑
11	Beta-citraurin	C30H40O2	HMDB35091	477.30	13.92	1.37	0.001	↑
12	Nervonic acid	C24H46O2	HMDB02368	411.35	12.39	1.44	0.001	↑
13	8-Keto palmitic acid	C16H30O3	LMFA01060055	539.43	12.59	1.48	0.001	↑

**Table 3:** Potential differential metabolites between the DKD group and NKK20 group.

No	Compounds	Formula	Library ID	m/z	R/T(min)	VIP	*P*	Change
1	Palmityl palmitate	C32H64O2	LMFA07010001	479.48	11.69	1.46	0.001	↓
2	Nonadecanoic acid	C19H38O2	HMDB00772	343.28	12.22	1.42	0.001	↓
3	DG(O-16 : 0/18 : 1(9Z))	C37H72O4	LMGL02020001	625.54	12.17	1.37	0.001	↓
4	15(S)-HETE	C20H32O3	HMDB03876	319.23	10.06	1.35	0.001	↑
5	N-docosahexaenoyl glutamic acid	C27H39NO5	LMFA08020089	502.28	9.53	1.31	0.001	↑
6	SM(d18 : 1/16 : 0)	C39H79N2O6P	LMSP03010003	747.56	9.68	1.30	0.001	↑
7	16-a-Hydroxypregnenolone	C21H32O3	HMDB00315	313.22	9.11	1.29	0.001	↓
8	PC(17 : 2(9Z,12Z)/0 : 0)	C25H48NO7P	LMGP01050127	504.31	9.57	1.29	0.001	↑
9	PC(18 : 1(9E)/0 : 0)	C26H52NO7P	LMGP01050030	566.34	10.21	1.28	0.001	↑
10	20-Oxo-leukotriene E4	C23H35NO6S	HMDB12642	452.21	9.97	1.27	0.001	↓
11	PC(16 : 0/0 : 0)	C24H50NO7P	LMGP01050018	494.32	10.50	1.24	0.001	↑
12	PC(O-18 : 1(1E)/0 : 0)	C26H54NO6P	LMGP01070008	552.37	10.42	1.24	0.001	↑
13	PC(O-16 : 0/0 : 0)	C24H52NO6P	LMGP01060010	526.35	10.25	1.21	0.001	↑
14	PC(19 : 3(10Z,13Z,16Z)/0 : 0)	C27H50NO7P	LMGP01050003	552.31	9.38	1.21	0.001	↑
15	PC(O-15 : 0/0 : 0)	C23H50NO6P	LMGP01060009	466.33	10.25	1.18	0.001	↑
16	SM(d16 : 1/17 : 0)	C38H77N2O6P	LMSP03010037	687.54	5.63	1.16	0.01	↑
17	13S-Hydroxyoctadecadienoic acid	C18H32O3	HMDB04667	295.23	9.75	1.13	0.01	↑
18	PC(18 : 2(9Z,12Z)/15 : 0)	C41H78NO8P	HMDB08132	742.54	11.66	1.12	0.01	↑
19	SM(d18:1/16 : 1)	C39H77N2O6P	LMSP03010041	745.55	10.74	1.12	0.01	↑
20	8-Keto palmitic acid	C16H30O3	LMFA01060055	539.43	12.59	1.10	0.01	↓
21	PC(20 : 4(5Z,8Z,11Z,14Z)/0 : 0)	C28H50NO7P	HMDB10395	588.33	9.42	1.08	0.01	↑

## Data Availability

The raw data supporting the conclusions of this article will be made available by the authors.
